# Adaptive-to-maladaptive IRE1α signaling as a driver of amyloidogenic APP processing in Alzheimer’s disease

**DOI:** 10.3389/fmolb.2026.1903849

**Published:** 2026-08-11

**Authors:** Daniel Wang, Qinan Yin

**Affiliations:** 1 Bullis School, Potomac, MD, United States; 2 Department of Radiation Oncology, Cancer Institute, The First Affiliated Hospital, and College of Clinical Medicine of Henan University of Science and Technology, Luoyang, Henan, China

**Keywords:** Alzheimer’s disease, amyloid-β, APP processing, BACE1, ER stress, IRE1α, RIDD, XBP1s

## Abstract

Alzheimer’s disease (AD) is increasingly recognized as a disorder of proteostatic failure characterized by progressive disruption of neuronal protein quality control, culminating in amyloid-β (Aβ) accumulation and synaptic dysfunction. Chronic activation of the endoplasmic reticulum (ER) stress response represents one of the earliest molecular alterations detected in vulnerable brain regions and correlates with Braak progression before overt plaque deposition. Inositol-requiring enzyme 1 alpha (IRE1α), the most evolutionarily conserved sensor of the unfolded protein response (UPR), functions as a signaling rheostat within this network through its divergent downstream outputs. Under moderate proteotoxic stress, adaptive IRE1α- X-box binding protein 1 (XBP1) signaling supports ER proteostasis, preserves amyloid precursor protein (APP) quality control, and favors non-amyloidogenic α-secretase processing. Persistent ER stress, however, drives sustained IRE1α hyperactivation and engages regulated IRE1α-dependent decay (RIDD), which destabilizes microRNA (miRNA) networks that normally constrain beta-site APP-cleaving enzyme 1 (BACE1) expression, thereby favoring amyloidogenic APP processing. Accumulating evidence suggests that aging progressively compromises ER proteostatic capacity, thereby redirecting IRE1α signaling away from adaptive XBP1s-mediated responses toward a predominantly RIDD-driven state. This shift may reinforce a self-sustaining cycle in which accumulating Aβ further amplifies ER stress signaling. Here, we examine how dynamic changes in IRE1α signaling bias contribute to amyloidogenic progression in AD and consider whether selective modulation of adaptive versus maladaptive IRE1α outputs may offer stage-dependent therapeutic benefit.

## 1 Introduction

AD affects more than 50 million individuals worldwide and remains the leading cause of dementia in the aging population (Li et al., 2022). Although amyloid accumulation is a defining pathological feature of AD, increasing evidence suggests that the disease cannot be fully explained as a disorder of protein deposition alone. Instead, AD is increasingly viewed as a progressive failure of neuronal proteostatic homeostasis that precedes extensive plaque formation and reshapes the intracellular environment during early disease progression (Hetz et al., 2020; Ciechanover and Kwon, 2017). APP may be particularly vulnerable to this proteostatic instability because relatively subtle changes in its trafficking or processing can substantially influence amyloidogenic burden (Ciechanover and Kwon, 2017).

Activation of the ER stress response is among the earliest molecular abnormalities detected in AD vulnerable brain regions. Postmortem studies have shown that UPR signaling markers, including phosphorylated IRE1α, PERK, and eukaryotic initiation factor 2 alpha (eIF2α), are elevated in hippocampal neurons across Braak stages and correlate with progressive neurofibrillary pathology (Hoozemans et al., 2009). Importantly, these alterations are detectable in neurons exhibiting early tau pathology before mature tangle formation, suggesting that ER stress activation may arise during early disease progression rather than exclusively as a secondary consequence of advanced neurodegeneration (Hashimoto and Saido, 2018). Similar findings have been observed in amyloid precursor protein/presenilin 1 (APP/PS1) transgenic models, where elevated expression of glucose-regulated protein 78 (GRP78), also known as binding immunoglobulin protein (BiP), together with enhanced XBP1 splicing, precedes overt amyloid plaque deposition (Gerakis and Hetz, 2018). These observations place ER proteostatic dysfunction early in the pathogenic cascade of AD.

Among the three principal UPR sensors, IRE1α occupies a central position because its downstream outputs exert divergent effects on neuronal proteostasis and APP processing. Under moderate proteotoxic stress, activation of the IRE1α XBP1s axis enhances ER folding capacity, supports ER associated degradation, and promotes adaptive restoration of proteostatic homeostasis (Hetz et al., 2020). Persistent ER stress, however, drives sustained IRE1α hyperactivation and engages regulated RIDD, a broader RNase activity capable of destabilizing transcripts and miRNA networks involved in BACE1 regulation (Adams et al., 2019; Hollien et al., 2009). The IRE1α–tumor necrosis factor receptor-associated factor 2 (TRAF2)–apoptosis signal-regulating kinase 1 (ASK1)—c-Jun N-terminal kinase (JNK) signaling axis has likewise been implicated in tau hyperphosphorylation, neuroinflammatory activation, and neuronal injury in AD (Hollien et al., 2009; Salminen et al., 2009). These observations suggest that IRE1α signaling exerts highly context dependent effects during disease progression.

The activating transcription factor 6 (ATF6) primarily supports adaptive ER stress recovery through transcriptional induction of molecular chaperones and protein-folding machinery. By contrast, IRE1α possesses dual kinase and endoribonuclease activities that enable dynamic switching between adaptive XBP1s signaling and maladaptive RIDD activation during AD progression. This functional duality allows IRE1α to initially support neuronal proteostasis yet later promote BACE1 derepression, APP mis-trafficking, and chronic amyloidogenic stress once higher-order phospho-IRE1α oligomerization becomes stabilized (Adams et al., 2019; Hollien et al., 2009; Salminen et al., 2009; Duran-Aniotz et al., 2017; Hébert et al., 2008).

Here, we examine how dynamic shifts in IRE1α signaling bias influence amyloidogenic APP processing during AD progression and consider whether selective preservation of adaptive IRE1α outputs may provide a stage-dependent strategy for restoring neuronal proteostasis. We focus specifically on the transition of IRE1α signaling from an adaptive to a maladaptive state during amyloidogenic APP processing. Although tau pathology is discussed where relevant, our framework is conceptualized to address this specific pathogenic axis, rather than to present a comprehensive model of Alzheimer’s disease pathogenesis.

## 2 Adaptive IRE1α-XBP1s signaling preserves APP proteostasis

In the early stages of AD pathogenesis, accumulation of Aβ oligomers and misfolded APP variants within the ER lumen activates an adaptive UPR centered on IRE1α-XBP1s signaling. Dissociation of GRP78 from the luminal domain of IRE1α permits IRE1α dimerization and activation of its RNase domain (Hetz et al., 2020). During early-stage AD, IRE1α activity remains biased toward XBP1 mRNA splicing rather than broad RIDD activation, thereby supporting restoration of ER homeostasis (Gerakis and Hetz, 2018). Postmortem analyses are consistent with this stage-dependent pattern. XBP1s mRNA is detectable in neurons with early tau pathology, whereas markers associated with RIDD activity become more prominent in advanced disease stages (Hoozemans et al., 2009). These observations suggest that early IRE1α-XBP1s activation initially functions as a compensatory response to emerging proteotoxic stress.

XBP1s induces ER chaperones including GRP78, glucose-regulated protein 94 (GRP94), and protein disulfide isomerase (PDI), which facilitate APP folding and maturation within the ER lumen (Hetz et al., 2020). GRP78 directly interacts with nascent APP and limits premature aggregation before Golgi trafficking. Reduced GRP78 function has been associated with impaired APP maturation and increased amyloidogenic processing in AD neurons (Ciechanover and Kwon, 2017). XBP1s also induces endoplasmic reticulum-associated degradation (ERAD) components including HMGCR degradation protein 1 (HRD1) and suppressor/enhancer of Lin-12-like protein 1 (SEL1L), which promote proteasomal clearance of misfolded APP variants. XBP1s-dependent induction of HRD1 has been shown to modulate BACE1 protein stability following Aβ42 oligomer exposure in SH-SY5Y neuroblastoma and APP-overexpressing CHO cell models, providing a mechanistic link between adaptive XBP1s signaling and regulation of amyloidogenic APP processing (Gerakis et al., 2016). IRE1 overexpression in *Drosophila* AD models reduces mutant APP accumulation through a mechanism requiring Hrd1 and the ribosome quality control (RQC)-associated ATPase Valosin-Containing Protein (VCP), also known as p97, demonstrating that IRE1-driven ERAD activity directly limits APP proteotoxicity independently of transcriptional XBP1s output (Li et al., 2024). Meanwhile, XBP1s transcriptionally upregulates a disintegrin and metalloproteinase 10 (ADAM10), the principal α-secretase involved in non-amyloidogenic APP cleavage (Reinhardt et al., 2014). Reduced XBP1s-dependent ADAM10 induction in AD tissue and APP-overexpressing models may therefore favor amyloidogenic APP processing (Reinhardt et al., 2014).

Studies in fibroblasts from patients with familial and sporadic AD, together with presenilin-mutant cell models, have shown that chronic ER stress disrupts mitochondria-associated ER membrane (MAM) organization and alters APP trafficking toward amyloidogenic processing pathways (Area-Gomez et al., 2012).

Additional evidence supports a protective role for the IRE1α-XBP1s axis in AD-related proteotoxicity. In *Drosophila*, XBP1s overexpression reduced Aβ-induced neurodegeneration and attenuated calcium-dependent ER stress through downregulation of ryanodine receptor 3 (RyR3) channels (Casas-Tinto et al., 2011). In *Caenorhabditis elegans* AD models, XBP1s overexpression enhanced Aβ clearance, whereas xbp-1 knockdown exacerbated Aβ-associated toxicity (Marcora et al., 2017). In 5xFAD mice, central nervous system-wide adeno-associated virus-mediated delivery of XBP1s reduced soluble and deposited amyloid-β burden, restored long-term potentiation, and improved hippocampal-dependent memory performance (Duran-Aniotz et al., 2023). Proteomic analysis further demonstrated normalization of protein networks associated with synaptic maintenance, autophagy, and endoplasmic reticulum-associated degradation, consistent with restoration of adaptive neuronal proteostasis (Duran-Aniotz et al., 2023). Similarly, chaperone intervention therapy that preferentially enhanced IRE1α-XBP1s signaling increased XBP1s and ADAM10 expression in APPNL-G-F knock-in mice and improved spatial learning, including at relatively late disease stages (Hafycz et al., 2023). These findings support a protective role for adaptive IRE1α-XBP1s signaling during early AD progression. Progressive loss of this adaptive capacity may facilitate the transition toward sustained amyloidogenic APP processing.

**FIGURE 1:**
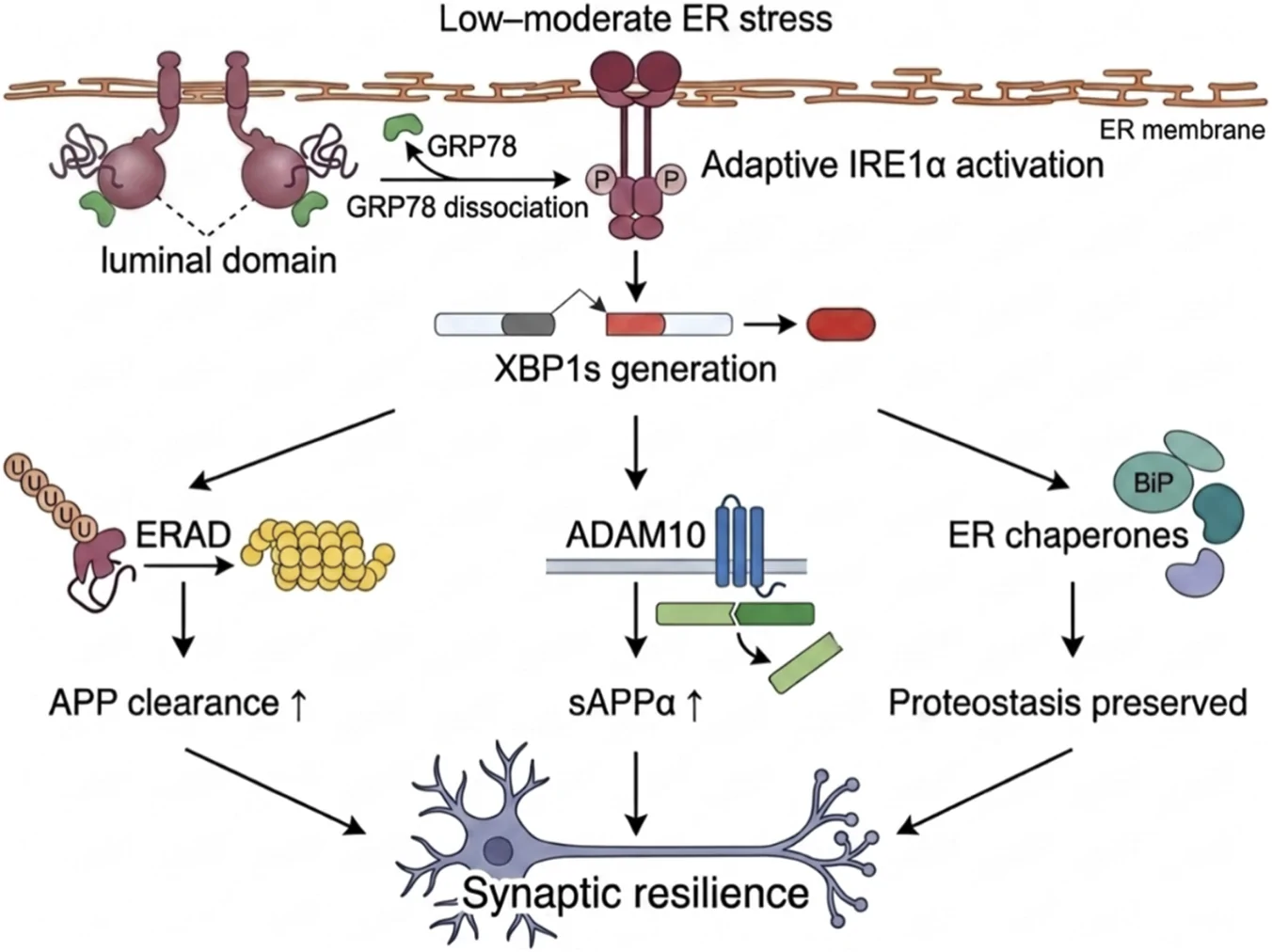
Proposed mechanism of adaptive IRE1α signaling under low-moderate ER stress. Under stress, GRP78 dissociates from the IRE1α luminal domain, initiating IRE1α activation and XBP1 mRNA splicing. The active transcription factor XBP1s induces ER chaperones to maintain proteostasis while concurrently stimulating ERAD-mediated APP clearance. Additionally, XBP1s upregulates ADAM10 activity, driving non-amyloidogenic APP processing and sAPPα production. Collectively, these responses preserve synaptic resilience and neuronal homeostasis during early-stage AD. This schematic summarizes adaptive IRE1α signaling based primarily on evidence from *Drosophila*, rodent, and cell-based studies. Several mechanisms remain supported mainly by experimental models, and the strength of evidence varies among the pathways illustrated.

These findings support a model in which adaptive IRE1α–XBP1s signaling preserves neuronal proteostasis during early AD progression by enhancing ER folding capacity, promoting ER-associated degradation, favoring non-amyloidogenic APP processing, and maintaining synaptic resilience under moderate proteotoxic stress. The proposed mechanistic framework underlying adaptive IRE1α signaling in preclinical AD is summarized in Figure 1.

## 3 Chronic IRE1α hyperactivation drives amyloidogenic APP processing

### 3.1 RIDD-dependent BACE1 derepression and feedforward amyloidogenesis

Current preclinical evidence supports the concept that the transition from adaptive to maladaptive IRE1α signaling represents a defining event in the progression of AD-associated proteostasis collapse. Whether this transition occurs in human AD remains uncertain. In vulnerable AD neurons, moderate ER stress initially promotes transient IRE1α dimerization and selective XBP1 mRNA splicing, thereby supporting ER folding capacity and adaptive stress buffering. Progressive Aβ42 accumulation within vulnerable neurons drives a feedforward deterioration of ER homeostasis in which sustained calcium leakage and oxidative misfolding collectively lower the threshold for stable higher-order phospho-IRE1α oligomerization, broadening RNase substrate selectivity beyond the selective XBP1 mRNA splicing characteristic of early adaptive responses (Ghosh et al., 2014). IRE1α signaling shifts away from adaptive XBP1s production toward sustained RIDD activation.

One major pathological consequence of sustained RIDD activation is progressive derepression of BACE1, the rate-limiting β-secretase responsible for amyloidogenic APP cleavage. BACE1 protein abundance and enzymatic activity are elevated in hippocampal and temporal cortical tissue from sporadic AD patients and correlate with amyloid burden and cognitive decline (Hampel et al., 2021). Chronic RIDD signaling has been proposed to destabilize miRNAs that normally suppress BACE1 translation, including miR-29a/b-1 and miR-107. Although reduced expression of these miRNAs correlates with elevated BACE1 levels in human postmortem AD tissue (Hébert et al., 2008; Wang et al., 2008), direct evidence for RIDD-mediated cleavage of these specific transcripts in human AD neurons remains lacking. The convergence of UPR branch activation and miRNA dysregulation in AD represents a broader regulatory network in which IRE1α-dependent and PERK-dependent signaling pathways each contribute to disruption of post-transcriptional BACE1 constraint through mechanistically distinct miRNA targets (Ajoolabady et al., 2022). Both miRNAs bind regulatory sequences within the BACE1 3′ untranslated region (UTR) and limit basal BACE1 translation under physiological conditions. In postmortem AD cortex, reduced miR-29a/b-1 expression inversely correlates with elevated BACE1 protein accumulation, whereas loss of miR-107 is detectable during early pathological stages preceding extensive plaque deposition (Hébert et al., 2008; Wang et al., 2008). Consistently with this, Aβ induced IRE1α activation in human neuronal cell models increases p-IRE1α levels and disrupts microRNA homeostasis, effects attenuated by pharmacological IRE1α RNase inhibition with STF-083010 (Li et al., 2019). Together, these findings implicate chronic RIDD signaling as a mechanistic contributor to amyloidogenic APP processing in AD-relevant stress contexts.

Chronic RIDD activation may further erode adaptive proteostatic capacity by targeting transcripts required for ER homeostasis. Notably, heat shock protein family A member 5 (HSPA5) mRNA, which encodes GRP78, has been identified as a potential RIDD substrate under conditions of sustained ER stress, suggesting that prolonged IRE1α activation may progressively compromise the chaperone network that initially buffers amyloidogenic stress (Gomez and Rutkowski, 2016). Progressive depletion of GRP78 reduces APP folding efficiency, increases accumulation of misfolded APP intermediates within the ER lumen, and lowers the threshold for persistent IRE1α activation. Impaired ER chaperone buffering additionally disrupts ER-associated degradation efficiency, further increasing retention of misfolded APP species within stressed AD neurons. As adaptive folding and degradation systems progressively collapse, neuronal proteostasis becomes increasingly dependent on chronic stress signaling pathways that further amplify amyloidogenic processing.

Aβ42 oligomers may further stabilize chronic IRE1α hyperactivation through calcium-dependent disruption of GRP78 buffering capacity and membrane lipid perturbations that destabilize the IRE1α transmembrane domain (Casas-Tinto et al., 2011; Volmer et al., 2013). Sustained activation of the IRE1α–XBP1 signaling axis disrupts mitochondria-associated ER membrane integrity, impairs mitochondrial membrane potential, and increases reactive oxygen species accumulation in Aβ-stressed human neuronal models. Pharmacological inhibition of IRE1α with 4μ8C partially reverses these abnormalities, supporting a mechanistic link between chronic IRE1α hyperactivation and mitochondrial dysfunction in AD-relevant neurons (Chu et al., 2021).

### 3.2 IRE1α-TRAF2-ASK1-JNK signaling disrupts APP trafficking and promotes tau pathology

Chronic IRE1α hyperactivation in AD neurons extends beyond RIDD-dependent BACE1 derepression and progressively engages the TRAF2-ASK1-JNK signaling axis, thereby coupling sustained ER stress to APP mis-trafficking, tau hyperphosphorylation, and cytoskeletal disorganization (Duran-Aniotz et al., 2017). During early adaptive stress responses, IRE1α activity remains largely biased toward XBP1 mRNA splicing with limited activation of downstream stress kinases. Prolonged Ser724 autophosphorylation stabilizes higher-order IRE1α oligomers that increasingly recruit TRAF2 to the cytoplasmic kinase domain of IRE1α (Ghosh et al., 2014). This conformational transition promotes sustained activation of ASK1 and persistent downstream JNK phosphorylation. In postmortem AD hippocampus, phosphorylated IRE1α increases progressively across Braak stages and colocalizes with neurons exhibiting early tau pathology, supporting the association between chronic IRE1α signaling and advancing neurodegeneration (Duran-Aniotz et al., 2017).

Sustained JNK activation alters APP trafficking through phosphorylation of APP at Thr668 within the APP cytoplasmic domain (Ando et al., 1999). Phosphorylated APP undergoes enhanced internalization from the plasma membrane into Rab5-positive early endosomes enriched in BACE1 activity, thereby increasing spatial coupling between APP and β-secretase processing machinery (Ando et al., 1999). In stressed AD neurons, this trafficking redistribution shifts APP away from non-amyloidogenic surface processing and toward amyloidogenic endosomal cleavage pathways. Chronic JNK signaling further disrupts axonal transport through phosphorylation of kinesin light chain (KLC), impairing anterograde trafficking of APP-containing vesicles (Mórotz et al., 2019). As transport efficiency progressively declines, APP accumulates within somatodendritic compartments where BACE1 abundance is relatively high, further amplifying intracellular amyloidogenic processing and Aβ generation.

The pathological consequences of sustained JNK activation extend beyond APP trafficking alone. Activated JNK phosphorylates tau at disease-associated epitopes including Thr231 and Ser202, modifications closely linked to reduced microtubule binding affinity and cytoskeletal destabilization (Duran-Aniotz et al., 2017). Progressive tau hyperphosphorylation impairs axonal integrity and disrupts intracellular transport of mitochondria, synaptic cargo, and proteostasis-related vesicles. These transport defects further increase intracellular retention of misfolded proteins and damaged organelles, thereby reinforcing ER stress signaling within vulnerable AD neurons.

These observations position the IRE1α–JNK signaling axis as a mechanistic interface linking chronic proteostatic stress to both amyloidogenic APP processing and tau-associated cytoskeletal disruption in AD neurons.

### 3.3 Calcium dysregulation and lipid membrane stress drive chronic IRE1α hyperactivation

As AD pathology progresses, sustained disruption of ER calcium homeostasis increasingly favors chronic IRE1α activation in vulnerable neurons. Transient calcium release from the ER supports chaperone-mediated protein folding and allows neurons to adapt to fluctuating proteostatic demand. In neurons exposed to persistent Aβ42 accumulation, however, this balance gradually deteriorates. Chronic activation of RyR3 and inositol 1,4,5-trisphosphate receptor channels promote continuous calcium leakage from the ER lumen (Casas-Tinto et al., 2011). Reduced luminal calcium impairs oxidative protein folding and weakens GRP78-dependent chaperone activity, allowing misfolded APP intermediates to accumulate within the ER. The resulting increase in proteotoxic burden further destabilizes ER homeostasis and prolongs stress signaling. *In vivo* multiphoton imaging of APP/PS1 mice confirmed that elevated mitochondrial calcium levels precede neuronal death and are induced by soluble Aβ oligomers at concentrations comparable to those found in human AD brain, with mitochondrial calcium uniporter blockade preventing this effect (Calvo-Rodriguez et al., 2020). Calcium depletion also alters the signaling behavior of IRE1α itself. During transient ER stress, reassociation of GRP78 with the IRE1α luminal domain helps terminate signaling and preserves RNase selectivity toward adaptive XBP1 mRNA splicing (Belyy et al., 2022). Prolonged luminal calcium depletion weakens this regulatory interaction and stabilizes higher-order phospho-IRE1α oligomers that preferentially support RIDD activity in chronically stressed AD neurons (Ghosh et al., 2014; Belyy et al., 2022). Aging further increases susceptibility to this transition because proteasomal efficiency and ER chaperone reserve decline before extensive plaque deposition becomes established (Naidoo, 2009). Under these conditions, signaling pathways that initially buffer proteotoxic stress gradually become difficult to disengage.

Membrane-associated stress provides an additional mechanism sustaining chronic IRE1α activation. Aβ42 oligomers alter ER membrane lipid organization, membrane curvature, and bilayer fluidity, changes that directly influence the conformational stability of IRE1α within the ER membrane (Volmer et al., 2013). It has been reported that increased membrane lipid saturation alone is sufficient to activate IRE1α independently of unfolded protein accumulation (Volmer et al., 2013).

Chronic IRE1α hyperactivation therefore emerges not from a single upstream lesion, but from the progressive convergence of calcium instability, membrane perturbation, and proteostatic exhaustion within vulnerable AD neurons.

**FIGURE 2:**
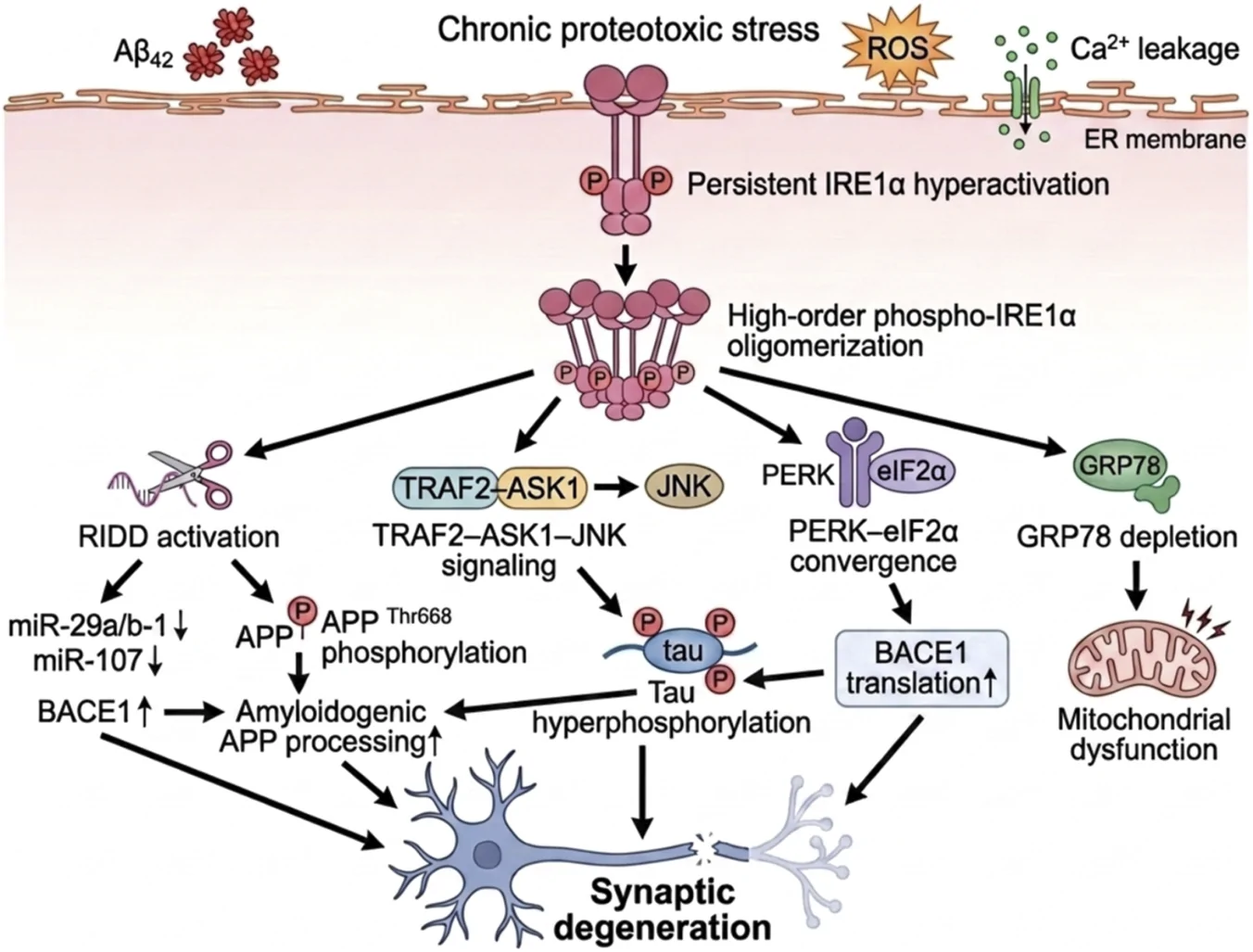
Chronic maladaptive IRE1α signaling promotes amyloidogenic APP processing and synaptic degeneration in advanced AD. Persistent IRE1α hyperactivation promotes formation of high-order phospho-IRE1α oligomers with expanded RNase and kinase signaling outputs. Sustained RIDD destabilizes miR-29a/b-1 and miR-107, relieving post-transcriptional repression of BACE1 and enhancing amyloidogenic APP processing. Concurrent activation of the TRAF2–ASK1–JNK axis promotes APP Thr668 phosphorylation and tau hyperphosphorylation, further impairing neuronal trafficking and cytoskeletal stability. Chronic PERK–eIF2α signaling converges with IRE1α pathways to enhance BACE1 translation, whereas depletion of GRP78 buffering capacity contributes to calcium dyshomeostasis and mitochondrial dysfunction. Together, these interconnected stress pathways establish a self-reinforcing feedforward network that amplifies proteotoxic stress and culminates in progressive synaptic degeneration during advanced AD. This schematic integrates maladaptive IRE1α signaling mechanisms reported across human postmortem tissue, rodent models, and cell-based systems. The strength of evidence varies among the pathways illustrated, with some mechanisms supported primarily by correlative observations in human tissue and others derived mainly from mechanistic studies in experimental models.

Collectively, these findings support a model in which chronic proteotoxic stress progressively stabilizes maladaptive IRE1α signaling networks involving sustained RIDD activation, kinase-driven APP mis-trafficking, calcium dyshomeostasis, and mitochondrial dysfunction. The integrated pathological framework is summarized in Figure 2.

## 4 Modulators of IRE1α signaling bias and a disease staging model

### 4.1 Oligomerization state and proteostatic thresholds determine IRE1α output specificity

While structural and biochemical studies in heterologous systems indicate that the transition from adaptive XBP1 splicing to maladaptive RIDD is governed by IRE1α oligomerization, whether this regulatory model operates similarly in AD-affected neurons remains an open question. We propose that the oligomeric state of IRE1α, together with the surrounding proteostatic landscape, serves as a major determinant of signaling output throughout AD progression. Under transient ER stress conditions, IRE1α predominantly forms low-order dimers whose RNase activity selectively processes XBP1 mRNA, thereby supporting ER-associated degradation, chaperone induction, and adaptive recovery of protein folding capacity (Belyy et al., 2022; Le Thomas et al., 2021). Under chronic exposure to Aβ42 oligomers and misfolded tau species, AD neurons progressively accumulate higher-order phospho-IRE1α oligomeric assemblies associated with broadened RNase substrate selectivity (Ghosh et al., 2014; Belyy et al., 2022). This conformational transition shifts signaling output away from adaptive XBP1 splicing toward broad RIDD-mediated degradation of transcripts required for neuronal proteostasis maintenance.

A critical determinant of this transition is the availability of GRP78 within the ER lumen. GRP78 binding normally constrains spontaneous IRE1α oligomerization through interactions with its luminal domain. Accumulation of misfolded APP, Aβ42 oligomers, and hyperphosphorylated tau progressively increases competition for GRP78 binding capacity, lowering the threshold for sustained IRE1α activation in AD neurons (Li et al., 2020; Hetz and Papa, 2018). Once GRP78 buffering becomes insufficient, phospho-IRE1α oligomers persist even after partial resolution of the initiating stress signal. This prolonged activation state favors chronic RIDD signaling and destabilization of transcripts involved in adaptive stress recovery.

The aging brain appears particularly vulnerable to this shift because neuronal proteostasis reserve is already compromised before overt plaque deposition develops. Proteasomal activity declines substantially during aging in rodent cortex and hippocampus, while inducible ER chaperone responses become progressively attenuated (Naidoo, 2009). Proteasome dysfunction in human AD brain occurs early and deepens with Braak staging, with snRNA-seq analysis revealing coordinate transcriptional downregulation of proteasome subunit genes and disruption of nuclear respiratory factor 1 (NRF1)-mediated regulatory pathways that normally sustain proteasome biogenesis in neurons (Jiang et al., 2025). These findings suggest that the transition from adaptive to maladaptive IRE1α signaling is not determined solely by Aβ burden itself, but also by the progressive erosion of neuronal buffering systems that normally restrain chronic IRE1α oligomerization.

### 4.2 Aging-associated post-translational remodeling biases IRE1α toward maladaptive signaling

Altered oligomerization dynamics alone do not fully account for the heightened susceptibility of aging neurons to maladaptive IRE1α signaling during AD progression. Long before widespread neuronal loss becomes histopathologically evident, vulnerable hippocampal and entorhinal cortical neurons reside within a chronically stressed intracellular environment characterized by mitochondrial dysfunction, intracellular Aβ42 accumulation, impaired mitophagic clearance, and sustained exposure to microglia-derived inflammatory cytokines. In pretangle neurons from Braak stage III–IV AD cortex, phospho-IRE1α accumulation colocalizes with markers of protein nitration and nitric oxide synthase activation, indicating that post-translational remodeling of UPR signaling emerges relatively early in disease progression rather than only during late-stage neurodegeneration (Cabral-Miranda et al., 2022). Against this background of sustained oxidative and nitrosative stress, S-nitrosylation of conserved cysteine residues within the IRE1α RNase domain suppresses adaptive XBP1 mRNA splicing while leaving RIDD activity comparatively preserved (Cabral-Miranda et al., 2022). The asymmetry is mechanistically plausible. XBP1 splicing requires highly constrained substrate docking geometry within the RNase domain, whereas RIDD substrates are recognized through a comparatively permissive stem-loop recognition mechanism that appears less sensitive to cysteine modification (Le Thomas et al., 2021; Cabral-Miranda et al., 2022). Rather than simply intensifying ER stress, these changes fundamentally reprogram the signaling architecture of the unfolded protein response.

That shift becomes consequential because adaptive and maladaptive IRE1α outputs remodel different components of the neuronal proteostasis network. During adaptive proteostatic stress responses, XBP1s-dependent transcription expands ER folding reserve through induction of GRP78, protein disulfide isomerase family members, ER-associated degradation components including the HRD1–SEL1L complex, and autophagy regulators required for clearance of incompletely folded APP intermediates (Hetz et al., 2020; Gerakis et al., 2016). Once S-nitrosylation suppresses adaptive XBP1 splicing, this compensatory network weakens. However, RIDD activity remains engaged under the same nitrosative conditions, consistent with its less restrictive substrate recognition requirements, and continues destabilizing transcripts required for stress recovery. What emerges is an asymmetric proteostatic state in which degradative outputs remain active while the machinery needed to restore neuronal homeostasis progressively deteriorates.

Evidence from aging and AD-related experimental systems supports this interpretation. Genetic disruption of adaptive IRE1α-XBP1s signaling impaired long-term potentiation and accelerated synaptic dysfunction before extensive neuronal loss became apparent in aged murine hippocampus (Cabral-Miranda et al., 2022). Conversely, AAV-mediated XBP1s overexpression restored dendritic spine density, normalized subsets of age-associated proteomic remodeling linked to synaptic maintenance and proteostasis regulation, and rescued hippocampal-dependent memory performance toward levels observed in younger animals (Cabral-Miranda et al., 2022). Similar protective effects have been reported in induced pluripotent stem cells (iPSC) derived neurons from familial AD donors exposed to chronic Aβ42 oligomer treatment, where restoration of XBP1s signaling improved ER-associated degradation efficiency and reduced intracellular accumulation of APP-derived fragments (Duran-Aniotz et al., 2023). ER stress-inducible protective factors, including mesencephalic astrocyte-derived neurotrophic factor (MANF), are elevated in postmortem brain tissue across both preclinical and symptomatic stages of AD (Zhang et al., 2024). In transgenic and AAV-mediated overexpression mouse models, sustained MANF upregulation has been shown to contribute to synapse loss through disruption of RNA-binding protein function, illustrating how molecular responses that are initially adaptive to proteotoxic stress can become drivers of neuronal dysfunction as disease burden increases (Zhang et al., 2024). These findings support a model in which adaptive IRE1α signaling functions less as a transient stress pathway and more as a long-term buffering system that preserves neuronal proteostatic resilience during aging.

The transition toward maladaptive signaling emerges when aging-associated post-translational remodeling converges with chronic AD proteotoxic stress. Aging neurons do not encounter Aβ42 from a position of proteostatic equivalence. Reduced chaperone reserve, impaired proteasomal throughput, and mitochondrial bioenergetic decline collectively lower the threshold at which intracellular Aβ42 transitions from a manageable folding burden to an irreversible proteotoxic insult. Calcium leakage through ryanodine receptor 3 channels persist longer. Oxidized proteins accumulate more rapidly. Recovery of luminal GRP78 binding becomes incomplete. Once formed under these conditions, phospho-IRE1α oligomers fail to disassemble between successive stress episodes, progressively lowering the activation threshold for RIDD-dominant signaling. A feedforward cycle then develops that differs fundamentally from acute ER stress responses. Because aging neurons enter each stress episode with diminished recovery capacity, every cycle of incomplete proteostatic restoration leaves behind a progressively larger residual pool of misfolded substrates. The system does not fully reset before the next insult arrives.

Neurons therefore do not enter AD progression from an equivalent biological baseline. Some retain sufficient adaptive IRE1α reserve to buffer chronic proteotoxic stress despite substantial amyloid burden, while others transition rapidly toward irreversible synaptic dysfunction once the RIDD-dominant state becomes thermodynamically stabilized. This framework generates a testable prediction. Neurons with the highest baseline expression of XBP1s-responsive proteostatic programs should exhibit greater resilience to equivalent amyloid exposure in postmortem AD brain tissue. Whether such resilience signatures overlap with established genetic modifiers of AD risk, including APOE4 and clusterin (CLU) variants that independently influence proteostatic capacity, remains unresolved. An affirmative answer would suggest that genetic variation in adaptive IRE1α reserve contributes directly to the clinical heterogeneity of AD independently of amyloid burden alone.

### 4.3 Cross-branch UPR integration amplifies maladaptive outcomes

During early proteostatic stress, moderate PERK activation transiently reduces ER folding burden through eIF2α phosphorylation and selective ATF4 translation (Liang et al., 2021). As this convergent stress burden exceeds the adaptive capacity of vulnerable hippocampal and entorhinal cortical neurons, sustained ATF4 induction drives CHOP expression, committing the PERK branch to a pro-apoptotic rather than a cytoprotective output (Liang et al., 2021).

The pathological significance of this transition lies in the convergence of PERK and IRE1α signaling on the same amyloidogenic machinery through mechanistically distinct regulatory layers (Ajoolabady et al., 2022). In AD neurons, PERK-eIF2α signaling enhances BACE1 translation by increasing ribosomal access to the structured 5′ UTR of BACE1 mRNA during stress-induced translational repression (Liang et al., 2021). At the same time, chronic RIDD signaling further amplifies BACE1 accumulation through post-transcriptional derepression mechanisms described above (Hébert et al., 2008; Wang et al., 2008). The combined effect substantially increases β-secretase activity and accelerates amyloidogenic APP processing beyond the level produced by either pathway independently.

Cross-branch integration becomes more damaging once downstream kinase cascades are engaged. Sustained oligomerization of phospho-IRE1α recruits TRAF2 and ASK1, leading to persistent JNK activation (Duran-Aniotz et al., 2017). JNK-dependent phosphorylation of APP at Thr668 drives its internalization from the plasma membrane into early endosomes, where the acidic luminal environment and concentrated BACE1 activity create conditions that strongly favor amyloidogenic over non-amyloidogenic cleavage (Ando et al., 1999). The same signaling axis phosphorylates tau at disease-associated epitopes including Thr231 and Ser202, impairing microtubule stability and disrupting kinesin-dependent axonal transport (Duran-Aniotz et al., 2017). PERK signaling further amplifies this environment through CHOP-dependent transcriptional remodeling that increases reactive oxygen species accumulation and lowers the threshold for mitochondrial permeability transition. These pathways therefore converge on shared pathological endpoints including APP mis-trafficking, intracellular Aβ42 accumulation, tau-associated transport collapse, and synaptic destabilization. The major upstream triggers, downstream substrates, and pathological consequences associated with adaptive and maladaptive IRE1α signaling in AD are summarized in Table 1.

**TABLE 1:** Mechanistic integration of adaptive and maladaptive IRE1α signaling in AD.

Upstream trigger	IRE1α-associated mechanism	Downstream substrate and effect	Pathological consequence	References
Misfolded APP accumulation	XBP1s-dependent ER-associated degradation activation	HRD1, SEL1L, GRP78 induction	Partial restoration of APP proteostasis	Gerakis and Hetz (2018), Duran-Aniotz et al. (2023)
Chronic Aβ42 oligomer stress	Higher-order phospho-IRE1α oligomerization	Expansion of regulated IRE1-dependent decay substrate selectivity	Loss of adaptive proteostatic buffering	Ghosh et al. (2014), Belyy et al. (2022), Le Thomas et al. (2021)
Persistent RIDD activation	Degradation of miR-29a/b-1 and miR-107	β-site APP-cleaving enzyme 1 derepression	Increased amyloidogenic APP processing	Hébert et al. (2008), Wang et al. (2008)
IRE1α-TRAF2-ASK1 complex formation	c-Jun N-terminal kinase activation	APP Thr668 phosphorylation and tau hyperphosphorylation	APP mistrafficking and microtubule instability	Duran-Aniotz et al. (2017), Ando et al. (1999)
Ryanodine receptor 3-mediated calcium leakage	Sustained IRE1α Ser724 autophosphorylation	Stabilization of chronic oligomeric signaling state	Feedforward ER stress amplification	Casas-Tinto et al. (2011), Ghosh et al. (2014)
Chronic PERK-eIF2α signaling	Translational derepression of BACE1 5′ untranslated region	Increased BACE1 protein synthesis	Synergistic enhancement of amyloidogenic flux	Liang et al. (2021)
Microglial cytokine release	NF-κB and JNK activation in neighboring neurons	Reinforcement of APP misprocessing and tau phosphorylation	Paracrine amplification of neuronal stress signaling	Duran-Aniotz et al. (2017), Liang et al. (2021)

Cross-cellular signaling further reinforces this pathological state. Cytokines including tumor necrosis factor alpha (TNF-α) and interleukin-1β (IL-1β) activate neuronal nuclear factor kappa B (NF- κB) and JNK signaling through receptor-mediated paracrine pathways, reinforcing the same APP mis-trafficking and tau phosphorylation endpoints driven by cell-autonomous IRE1α and PERK hyperactivation in activated microglia surrounding extracellular Aβ plaques (Nunes et al., 2012; Grandjean et al., 2020). Astrocytic inflammatory signaling further increases oxidative and metabolic stress within vulnerable neuronal populations. The result is not a single linear pathway, but a redundant systems-level network in which intracellular proteotoxic stress and non-cell-autonomous inflammatory signaling continuously reinforce one another. This mechanistic redundancy accounts for the incomplete rescue observed when individual UPR branches are targeted therapeutically in AD models, even after substantial reduction of upstream amyloid burden.

Quantitative immunoblotting and immunohistochemical analyses of postmortem lateral temporal cortex from AD patients demonstrated that phospho-PERK accumulation correlates progressively with advancing Braak neurofibrillary staging and associates more strongly with tau pathology than with amyloid burden, while postsynaptic density protein 95 (PSD-95) levels decline in parallel with disease severity (Buchanan et al., 2020). These observations indicate that the pathological UPR state in AD is not simply the sum of individual branch activation, but a network property that emerges from cross-branch convergence on shared substrates and becomes self-sustaining once downstream kinase cascades and inflammatory amplification loops are engaged.

### 4.4 A three-stage model of IRE1α signaling bias across AD progression

Our synthesis of current experimental and neuropathological evidence supports a stage-dependent model in which IRE1α signaling shifts from adaptive proteostatic compensation toward chronic maladaptive amplification as neuronal buffering capacity progressively declines. This tripartite framework should be viewed as a conceptual model integrating findings across multiple experimental systems rather than as a longitudinally validated clinical staging paradigm. APP/PS1 mouse hippocampus, elevated XBP1 spliced isoform expression is detectable before dense-core plaque deposition becomes histologically apparent (Gerakis and Hetz, 2018), indicating that early IRE1α activation initially functions as a compensatory response to rising intracellular proteotoxic stress rather than as a marker of irreversible degeneration. During this early phase, low-order IRE1α dimers preferentially catalyze XBP1 mRNA splicing instead of broad RIDD activity (Belyy et al., 2022; Le Thomas et al., 2021). The resulting XBP1s transcriptional program maintains proteostatic buffering capacity above the threshold required for ER homeostasis recovery, and adaptive resolution continues to outpace proteotoxic accumulation (Hetz et al., 2020; Gerakis et al., 2016).

As proteotoxic stress persists, adaptive buffering capacity progressively declines while RIDD-dominant signaling becomes increasingly stabilized. The earliest molecular signature of this transition is the degradation of miR-29a/b-1 and miR-107, which relieves post-transcriptional repression of BACE1 and initiates a feedforward increase in amyloidogenic APP processing before frank synaptic dysfunction becomes histologically apparent (Hébert et al., 2008; Wang et al., 2008). Concurrently, progressive Ser724 autophosphorylation stabilizes higher-order IRE1α oligomers that increasingly recruit TRAF2, shifting kinase output toward sustained JNK activation and APP Thr668 phosphorylation (Duran-Aniotz et al., 2017; Ghosh et al., 2014). These events redirect APP from non-amyloidogenic surface processing toward BACE1-enriched endosomal compartments, amplifying intracellular Aβ42 accumulation at a stage when partial proteostatic buffering capacity remains biologically present but is no longer sufficient to prevent progressive amyloidogenic commitment.

Chronic RIDD activity, persistent kinase activation, calcium dyshomeostasis, and mitochondrial dysfunction do not operate as independent pathological processes in advanced AD but converge into a self-reinforcing network that sustains amyloidogenic signaling beyond the point at which any single lesion could be individually corrected. Human postmortem AD cortex demonstrates near-universal phospho-IRE1α accumulation within vulnerable hippocampal neurons at advanced Braak stages (Hoozemans et al., 2009), consistent with stabilization of this chronic maladaptive state during late-stage disease.

**FIGURE 3:**
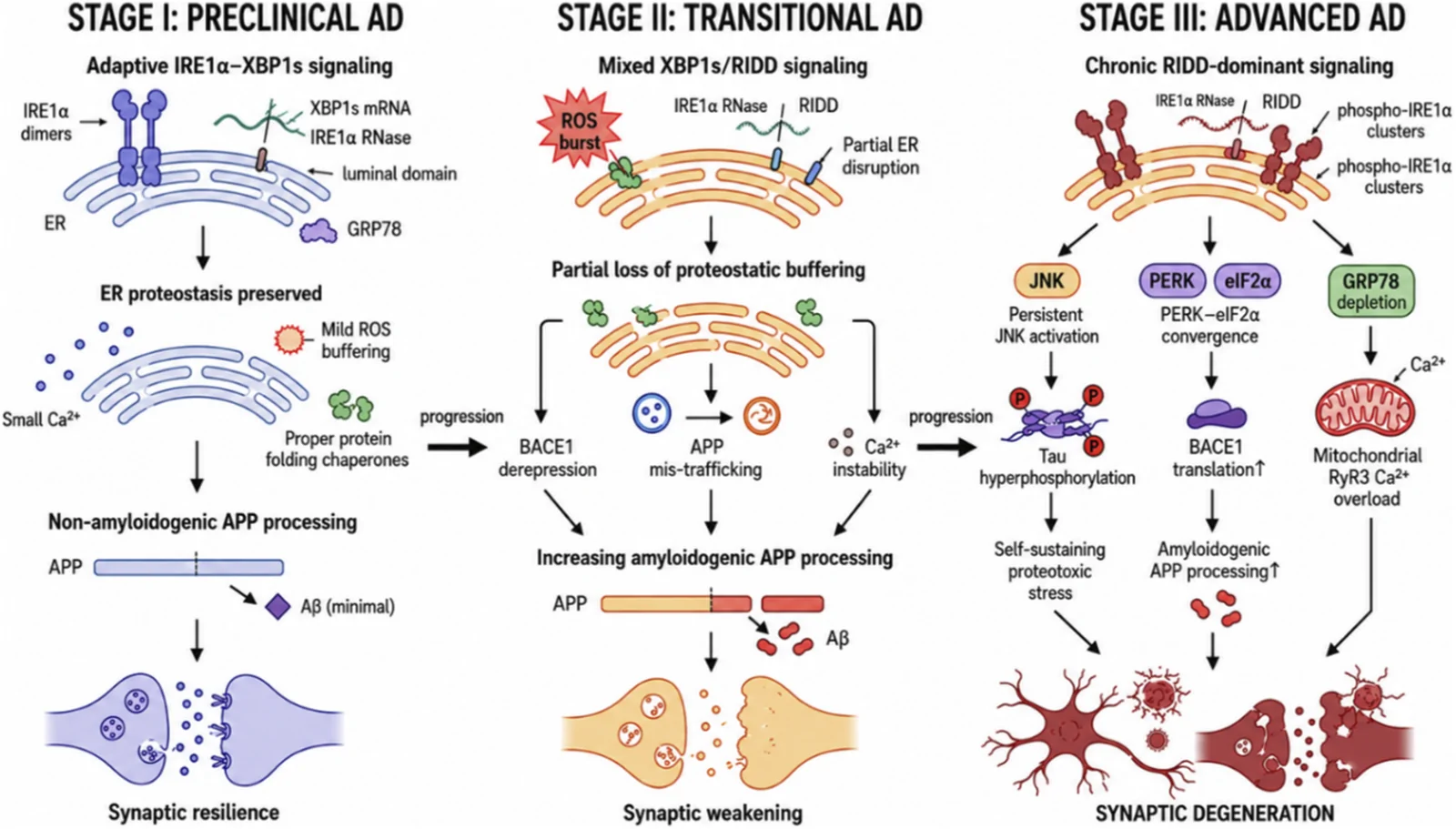
Stage-dependent changes in IRE1α signaling during AD progression. In early-stage AD, adaptive IRE1α–XBP1s signaling helps maintain ER proteostasis, supports non-amyloidogenic APP processing, and preserves synaptic function under moderate proteotoxic stress. As disease progression continues, increasing proteostatic burden and declining ER buffering capacity promote a transitional state characterized by mixed XBP1s and RIDD signaling, together with BACE1 derepression, APP mis-trafficking, calcium instability, and progressive amyloidogenic APP processing. In advanced AD, chronic RIDD-dominant signaling becomes stabilized and is accompanied by persistent JNK activation, PERK–eIF2α signaling, tau hyperphosphorylation, mitochondrial dysfunction, and sustained proteotoxic stress, ultimately contributing to irreversible synaptic degeneration. The model summarizes the progressive shift from adaptive proteostatic buffering to maladaptive stress signaling across AD stages. This staged model represents a conceptual framework synthesized from independent experimental studies. The temporal sequence and reversibility of the proposed stages have not been established in human AD.

This staging framework predicts that relative abundance of XBP1s-responsive transcripts compared with RIDD-sensitive targets in cerebrospinal fluid-derived neuronal exosomes represents a candidate biomarker for distinguishing adaptive from maladaptive ER stress states during AD progression. It further predicts that preservation of adaptive IRE1α signaling requires intervention before chronic phospho-IRE1α oligomerization, kinase-driven APP mis-trafficking, and proteostatic exhaustion become self-reinforcing. The stage dependent molecular and functional characteristics of adaptive-to-maladaptive IRE1α signaling across AD progression are summarized in Table 2. To conceptually integrate the progressive transition from adaptive XBP1s-dominant signaling to chronic RIDD-driven proteostatic collapse across AD progression, a stage-oriented mechanistic model is presented in Figure 3.

**TABLE 2:** Stage dependent IRE1α signaling bias across AD progression.

Disease stage	Dominant IRE1α state	Key molecular features	APP processing status	Functional consequence	References
Preclinical AD Braak stages I–III	Adaptive XBP1s-dominant low-order dimers	GRP78 induction, ERAD activation, autophagic buffering, preserved calcium homeostasis	Predominantly non-amyloidogenic APP processing	Reversible ER stress and preserved synaptic resilience	Gerakis and Hetz (2018), Duran-Aniotz et al. (2023), Belyy et al. (2022), Le Thomas et al. (2021)
Transitional AD Mild cognitive impairment to early symptomatic disease	Mixed XBP1s/RIDD signaling with progressive oligomer stabilization	miR-29a/b-1 and miR-107 degradation, BACE1 derepression, PERK-eIF2α activation, APP Thr668 phosphorylation	Increasing amyloidogenic APP trafficking and intracellular Aβ42 accumulation	Partial proteostatic exhaustion and unstable calcium homeostasis	Hébert et al. (2008), Casas-Tinto et al. (2011), Ghosh et al. (2014), Wang et al. (2008), Ando et al. (1999)
Advanced AD Braak stages V–VI	Chronic RIDD-dominant high-order phospho-IRE1α oligomers	Persistent JNK activation, tau hyperphosphorylation, mitochondrial dysfunction, chronic calcium dyshomeostasis	Self-sustaining amyloidogenic APP processing	Irreversible proteostatic collapse and synaptic degeneration	Hoozemans et al. (2009), Duran-Aniotz et al. (2017), Ando et al. (1999), Liang et al. (2021)

### 4.5 Context-dependent and disease-specific variability in IRE1α/XBP1 function

The stage-dependent model proposed above should be interpreted within the broader biological context of IRE1α signaling, in which neither XBP1s activation nor RIDD has inherently protective or pathological properties. Under physiological conditions, RIDD contributes to ER homeostasis by selectively degrading transcripts entering the secretory pathway, thereby limiting protein-folding burden in highly secretory cells. Its biological consequences are therefore determined by tissue type and the nature of the underlying stress rather than by RIDD activity itself (Maurel et al., 2014). RIDD appears to acquire pathogenic significance only after prolonged disruption of proteostasis in AD, suggesting that its biological consequences are dictated by the cellular environment rather than by fixed properties of the pathway itself.

A similar context dependence is evident for XBP1s signaling. Although activation of the IRE1α–XBP1s axis is consistently neuroprotective in experimental models of AD, the opposite has been reported in other neurodegenerative diseases. In SOD1 mouse models of amyotrophic lateral sclerosis, XBP1 deficiency enhances autophagic clearance of mutant SOD1 and prolongs survival (Hetz et al., 2009). Likewise, neuronal XBP1 silencing reduces mutant huntingtin accumulation and ameliorates disease progression in Huntington’s disease through FoxO1-dependent induction of autophagy (Vidal et al., 2012). Rather than representing conflicting observations, these studies suggest that the biological consequences of IRE1α signaling are shaped by the dominant proteostatic demands of each disease.

The duration of ER stress provides another important determinant of signaling outcome. Acute ER stress is generally accompanied by transient IRE1α activation that resolves once proteostasis is restored, whereas chronic stress favors persistent IRE1α oligomerization and sustained RNase activity (Li et al., 2024; Chu et al., 2021; Hetz and Papa, 2018). Collectively, these observations support a stage-dependent model of IRE1α signaling in AD while emphasizing that its biological consequences remain disease specific.

### 4.6 Cell-type specificity of IRE1α signaling in the AD brain

Although the mechanistic framework discussed here is derived largely from neuron-focused studies, IRE1α signaling also extends beyond neurons. In APP/PS1 mice, pharmacological inhibition of IRE1α with KIRA6 suppresses microglial activation and NF-κB signaling while reducing amyloid burden, supporting a role for microglial IRE1α in amplifying neuroinflammatory responses (Duran-Aniotz et al., 2017). These observations point to mechanisms that are distinct from the neuron-intrinsic regulation of APP processing and RIDD described above, suggesting that IRE1α contributes to AD through multiple cellular compartments rather than through neurons alone.

The contribution of astrocytes appears more difficult to interpret. CNS-wide XBP1s overexpression reduces astrogliosis together with improvements in neuronal proteostasis and amyloid pathology in 5xFAD mice (Duran-Aniotz et al., 2023), but whether this reflects direct regulation of astrocyte biology or simply a secondary consequence of reduced neuronal stress remains unresolved. Comparable information is largely lacking for cerebrovascular endothelial cells and oligodendrocytes, despite increasing recognition that vascular dysfunction, white matter abnormalities, and myelin loss contribute substantially to cognitive decline during AD progression.

This cellular heterogeneity is also relevant when considering therapeutic intervention. Systemically administered IRE1α modulators are likely to engage multiple CNS cell populations, making the overall response dependent on the balance of neuronal, glial, and vascular effects rather than on neuron-specific mechanisms alone. As more selective delivery technologies become available, targeting IRE1α signaling in specific cell populations may provide a more effective strategy than uniform modulation throughout the brain.

## 5 Therapeutic targeting of IRE1α signaling in AD

### 5.1 Global suppression of IRE1α RNase activity

Initial therapeutic efforts targeting IRE1α in AD emerged from the observation that chronic RIDD signaling amplifies amyloidogenic APP processing while destabilizing transcripts required for adaptive proteostasis in stressed neurons. Small-molecule inhibitors including KIRA6, STF-083010, and 4μ8C were therefore developed to suppress maladaptive IRE1α outputs through distinct mechanisms targeting either kinase-dependent oligomerization or RNase catalytic activity (Duran-Aniotz et al., 2017). KIRA6 acts as an allosteric kinase inhibitor that stabilizes IRE1α in a low-order conformation, indirectly suppressing both RIDD and XBP1 splicing activity, whereas STF-083010 and 4μ8C directly target the RNase domain and produce broader suppression of endonuclease activity independent of oligomerization state (Duran-Aniotz et al., 2017; Ghosh et al., 2014). In APP/PS1 mice, KIRA6 reduced hippocampal microglial activation, attenuated NF-κB signaling, and significantly lowered amyloid plaque burden within vulnerable hippocampal regions (Duran-Aniotz et al., 2017). Comparable anti-inflammatory effects were subsequently observed in Aβ42-treated neuronal cultures and SH-SY5Y cells exposed to chronic proteotoxic stress (Li et al., 2019).

That therapeutic logic later proved incomplete. STF-083010 and 4μ8C suppress RIDD activity, but they also block XBP1 mRNA splicing, thereby disrupting the adaptive branch of IRE1α signaling that maintains residual proteostatic capacity in vulnerable AD neurons (Duran-Aniotz et al., 2017). That distinction is most consequential during intermediate disease stages, when incomplete adaptive buffering persists despite chronic proteotoxic stress. Suppression of XBP1s signaling therefore erodes proteostatic capacity on multiple fronts simultaneously: molecular chaperone induction falters, ER-associated degradation becomes insufficient to clear misfolded substrates, and incompletely folded APP intermediates that would otherwise be retrotranslocated instead accumulate and enter amyloidogenic processing pathways. Broad IRE1α RNase inhibition in Aβ42-stressed primary neurons attenuated inflammatory signaling yet failed to restore APP proteostasis or improve Aβ clearance, suggesting that suppressing IRE1α output is not equivalent to recovering the adaptive signaling capacity that chronic stress has eroded (Li et al., 2019). The central therapeutic problem in AD is therefore not excessive IRE1α activity alone, but loss of signaling selectivity. Global inhibition suppresses maladaptive RIDD outputs while simultaneously dismantling the adaptive buffering systems that remain necessary for neuronal survival under chronic ER stress conditions.

### 5.2 Selective enhancement of adaptive XBP1s signaling

Recognition of this signaling asymmetry has shifted therapeutic interest toward selective preservation or enhancement of adaptive XBP1s signaling. Unlike broad RNase inhibition, this strategy attempts to maintain low-order IRE1α activity and preserve transcriptional programs that support APP folding, ER-associated degradation, autophagy, and synaptic maintenance. In 5xFAD mice with nervous-system-wide XBP1s overexpression, Aβ burden was reduced by 30%–40% in both cortex and hippocampus, and AAV-mediated hippocampal delivery of XBP1s independently restored long-term potentiation and improved hippocampal-dependent memory performance (Duran-Aniotz et al., 2023). Proteomic analysis further demonstrated correction of large subsets of age-associated synaptic and proteostatic remodeling (Duran-Aniotz et al., 2023). Whether XBP1s enhancement can similarly restore adaptive chaperone capacity and reduce proteotoxic vulnerability in iPSC-derived neurons from familial AD donors remains to be directly tested, but available evidence from Aβ-stressed human neuronal models supports the feasibility of this approach (Arber et al., 2020).

Selective pharmacological enhancement of XBP1s signaling has also become increasingly feasible. IXA4, a small-molecule activator of adaptive IRE1α signaling identified through high-throughput screening, enhances IRE1α RNase activity toward XBP1 mRNA without inducing RIDD-mediated transcript degradation or JNK phosphorylation, and improves ER proteostasis of destabilized APP variants through an IRE1-dependent mechanism in cellular models (Grandjean et al., 2020). This distinction is mechanistically important because XBP1 mRNA splicing requires precise substrate docking geometry within low-order IRE1α dimers, whereas RIDD activity emerges preferentially within higher-order oligomeric assemblies that broaden substrate selectivity (Belyy et al., 2022; Le Thomas et al., 2021). IXA4 therefore exploits this oligomeric state-dependent substrate hierarchy to preserve the adaptive conformational state of IRE1α rather than suppressing the pathway entirely.

Therapeutic effects of XBP1s enhancement may also extend beyond amyloidogenic APP processing. In a *Caenorhabditis elegans* tauopathy model, neuronal XBP1s activation reduced pathological tau accumulation and improved proteostatic resilience through coordinated regulation of chaperone and lipid metabolism gene networks (Waldherr et al., 2024; Waldh et al., 2019). These observations support that adaptive IRE1α signaling intersects simultaneously with APP proteostasis, tau-associated transport integrity, and membrane homeostasis. XBP1s-directed interventions may therefore target a broader pathological network than strategies focused exclusively on extracellular amyloid removal.

### 5.3 Upstream proteostasis stabilization and chemical chaperones

Chemical chaperones such as tauroursodeoxycholic acid (TUDCA) and 4-phenylbutyric acid (4-PBA) reduce accumulation of misfolded ER client proteins while partially restoring luminal folding capacity, lowering the proteotoxic threshold at which Aβ42-driven calcium dysregulation and membrane lipid perturbation would otherwise stabilize chronic phospho-IRE1α oligomerization and RIDD-dominant signaling (Casas-Tinto et al., 2011; Volmer et al., 2013; Nunes et al., 2012).

Findings from AD models support this framework. In APPNL-G-F knock-in mice, 4-PBA increased XBP1s expression across hippocampal regions including CA1, CA3, and dentate gyrus, reduced protein kinase RNA-like ER kinase phosphorylation, elevated ADAM10 abundance, and improved spatial learning and memory performance (Hafycz et al., 2023). TUDCA produced similar effects in APP/PS1 mice, reducing Aβ deposition and attenuating amyloidogenic amyloid precursor protein processing while suppressing mitochondrial apoptotic signaling linked to chronic ER stress (Nunes et al., 2012). Critically, neither agent directly suppresses IRE1α RNase activity, preserving residual adaptive XBP1s outputs while raising the proteotoxic threshold required to sustain maladaptive oligomerization.

This distinction has practical therapeutic implications. Both TUDCA and 4-PBA are already FDA-approved for non-neurological indications and possess blood-brain barrier permeability, features that substantially improve translational feasibility compared with more selective experimental IRE1α modulators. Their primary therapeutic value therefore lies less in suppressing IRE1α directly than in restoring the proteostatic conditions that normally prevent chronic RIDD-dominant signaling from becoming self-sustaining.

### 5.4 Translational challenges and biomarker-guided intervention

The most consequential unresolved challenge is therapeutic timing. Evidence from 5xFAD, and APPNL-G-F models indicates that adaptive IRE1α signaling remains partially recoverable during intermediate disease stages, before chronic phospho-IRE1α oligomerization and kinase-driven amplification loops become stabilized (Duran-Aniotz et al., 2023; Hafycz et al., 2023). Once persistent JNK activation, mitochondrial dysfunction, calcium dyshomeostasis, and maladaptive RIDD signaling become integrated into a self-reinforcing network, reduction of extracellular amyloid burden alone is insufficient to restore neuronal proteostasis (Hafycz et al., 2023; Scheper and Hoozemans, 2015). This framework offers a plausible mechanistic explanation for why lecanemab and donanemab substantially reduce plaque burden yet produce comparatively modest cognitive benefit in patients with established symptomatic AD (van Dyck et al., 2023; Sims et al., 2023).

Beyond therapeutic timing, translating IRE1α-targeted interventions into clinical settings faces several important pharmacological challenges. Foremost among these is chemical selectivity. KIRA6, a widely used kinase-inhibiting RNase attenuator, exhibits a broader off-target profile than originally appreciated. Photoaffinity-based chemoproteomic profiling revealed extensive cell-type-independent interactions between KIRA6 and multiple ATP-binding proteins (Korovesis et al., 2020). In addition, KIRA6 directly inhibits the receptor tyrosine kinase KIT (Mahameed et al., 2019) and suppresses inflammatory signaling through an IRE1α-independent interaction with HSP60 (Rufo et al., 2022), suggesting that some biological effects attributed to IRE1α inhibition may partly reflect off-target activities. Another major challenge concerns central nervous system (CNS) pharmacokinetics. Blood-brain barrier penetration and CNS exposure have not been systematically characterized for KIRA6, STF-083010, or 4μ8C. Likewise, although IXA4 selectively activates the IRE1α–XBP1s branch without inducing broader UPR activation, available pharmacological studies indicate predominantly hepatic distribution following systemic administration, and brain exposure has not yet been established (Grandjean et al., 2020). Long-term pharmacological modulation of IRE1α signaling also raises unresolved safety considerations. Because physiological IRE1α signaling contributes to normal proteostatic and immune homeostasis, prolonged manipulation of this pathway could potentially alter immune function or age-associated proteostatic capacity. Consistent with this possibility, pharmacological inhibition of IRE1α with 4μ8C selectively impairs IL-5 secretion in differentiated TH2 cells, supporting a role for IRE1α signaling in adaptive immune regulation (Poe et al., 2019). Collectively, these translational challenges do not diminish the mechanistic rationale for targeting IRE1α but instead highlight the need for rigorous chemoproteomic profiling, CNS pharmacokinetic characterization, and long-term safety evaluation before clinical translation.

Early translational development of IRE1α-directed therapy will likely require a staged clinical framework rather than immediate efficacy-driven intervention. Phase I studies should prioritize biomarker validation and target engagement, particularly quantification of XBP1s-responsive transcripts and RIDD-sensitive RNA signatures in cerebrospinal fluid-derived neuronal exosomes under defined disease-stage conditions (Goetzl et al., 2015). Phase II trials could then enrich for patients demonstrating preserved adaptive UPR buffering capacity, where restoration of low-order IRE1α signaling remains biologically recoverable (Duran-Aniotz et al., 2023; Hafycz et al., 2023). By contrast, Phase III studies will likely require combination strategies integrating IRE1α-directed modulation with established amyloid-lowering immunotherapies such as lecanemab or donanemab in order to determine whether simultaneous restoration of intracellular proteostatic resilience and extracellular amyloid clearance produces superior cognitive stabilization compared with amyloid removal alone (van Dyck et al., 2023; Sims et al., 2023). This framework recognizes that therapeutic responsiveness in AD is determined not only by amyloid burden, but also by the residual adaptive capacity of the neuronal proteostasis network. The stage-specific therapeutic rationale, biomarker strategies, and translational objectives associated with IRE1α-directed intervention in AD are summarized in Table 3. This biomarker strategy remains hypothetical at present and will require validation in longitudinal human cohort studies before clinical implementation can be considered.

**TABLE 3:** Stage-specific translational framework for IRE1α-Directed therapeutic intervention in AD.

Disease stage	Dominant pathological state	Therapeutic strategy	Biomarker focus	Translational objective	Representative therapeutic modality	References
Preclinical AD	Adaptive XBP1s-dominant signaling with preserved proteostatic reserve	Enhance adaptive IRE1α-XBP1s signaling	Increased XBP1s-responsive transcripts in cerebrospinal fluid-derived neuronal exosomes	Preserve neuronal proteostasis and delay transition toward maladaptive signaling	XBP1s gene delivery, IXA4-mediated adaptive IRE1α activation	Duran-Aniotz et al. (2023), Grandjean et al. (2020)
Early prodromal AD/Mild cognitive impairment	Mixed adaptive and maladaptive signaling with emerging RIDD activation	Stabilize low-order IRE1α signaling and restore ER buffering capacity	Relative ratio of XBP1s-associated transcripts to RIDD-sensitive RNA species	Prevent stabilization of chronic phospho-IRE1α oligomers	TUDCA, 4-PBA, adaptive IRE1α modulators	Hafycz et al. (2023), Nunes et al. (2012)
Symptomatic AD	Chronic RIDD-dominant signaling with kinase-driven amplification loops	Suppress maladaptive RIDD and inflammatory signaling	Phospho-IRE1α accumulation, RIDD-associated transcript depletion, neuroinflammatory signatures	Interrupt feedforward ER stress amplification	KIRA6, selective RNase modulation strategies	Duran-Aniotz et al. (2017), Hébert et al. (2008), Ghosh et al. (2014)
Advanced symptomatic AD	Self-sustaining proteostatic collapse and chronic intracellular stress remodeling	Combination therapy integrating proteostasis restoration with amyloid clearance	Longitudinal biomarker monitoring of adaptive signaling recovery during treatment	Improve cognitive stabilization beyond extracellular plaque reduction alone	Lecanemab or donanemab combined with IRE1α-directed intervention	van Dyck et al. (2023), Sims et al. (2023)

## 6 Discussion

Preclinical evidence supports a conceptual framework in which IRE1α functions as a context-dependent signaling hub governed by neuronal proteostasis rather than as a pathway requiring uniform suppression or activation (Duran-Aniotz et al., 2017; Cabral-Miranda et al., 2022). While the applicability of this framework to human Alzheimer’s disease remains to be established, findings from murine and other experimental models indicate that loss of adaptive IRE1α signaling accelerates cognitive decline during aging, whereas chronic phospho-IRE1α oligomerization promotes amyloidogenic APP processing (Duran-Aniotz et al., 2017; Cabral-Miranda et al., 2022). The central therapeutic problem may therefore lie less in absolute pathway activity than in progressive loss of signaling selectivity. Interventions that partially reduce amyloid burden tend to produce stronger biological effects during preclinical or prodromal stages of AD. Their diminished efficacy in advanced disease likely reflects not a loss of therapeutic potency, but the fact that the intracellular proteostatic network has already undergone extensive pathological remodeling by the time symptomatic neurodegeneration becomes established. Whether the degree of residual adaptive proteostatic reserve can be reliably quantified in living patients before irreversible IRE1α oligomerization becomes established remains the central unanswered question for translating this framework into clinical practice.

A central limitation of the current framework is that endogenous IRE1α signaling dynamics have not yet been directly resolved in vulnerable human neurons. Most mechanistic evidence derives from transgenic mouse lines, AAV overexpression systems, or *in vitro* neuronal models that do not fully recapitulate the cellular heterogeneity or slow temporal progression of sporadic AD. Whether the oligomerization-state-dependent output model proposed here accurately reflects endogenous IRE1α behavior across hippocampal and entorhinal cortical neuronal subtypes during progressive Braak staging remains unknown and will require single-nucleus transcriptomic and translatomic analysis of postmortem human AD tissue (Brase et al., 2023). Another unresolved problem is the RIDD substrate landscape itself. Beyond miR-29a/b-1, miR-107, and 14-3-3θ, the broader repertoire of RIDD-sensitive transcripts in human AD neurons has not been systematically characterized, leaving the full functional consequence of chronic RIDD activation incompletely understood (Gómora-García et al., 2021). It also remains unclear whether genetic modifiers beyond ATP-binding cassette transporter A7 (ABCA7) and XBP1 polymorphisms alter the long-term stability of adaptive IRE1α signaling during aging. Polygenic risk score analysis integrated with human brain RNA-sequencing has identified ER and Golgi secretory pathway genes among the disrupted biological processes enriched in both high-risk and diagnosed AD individuals, suggesting that variation in ER proteostatic networks contributes to AD genetic architecture beyond individual susceptibility loci (Crawford et al., 2023). That question matters because the clinical heterogeneity of AD suggests that neurons do not enter chronic proteotoxic stress with equivalent proteostatic resilience, even under comparable amyloid burden.

Future progress will depend on longitudinal biomarker profiling capable of resolving IRE1α signaling bias in living patients and on CRISPR-based perturbation studies in human iPSC-derived neuronal systems under physiologically relevant stress conditions. While this framework was developed specifically in the context of amyloidogenic APP processing, its applicability to tau-dominant or mixed-pathology dementias remains an important question for future investigation.

The relationship between IRE1α signaling and tau pathology appears to be more complex than a simple downstream consequence of altered APP processing. As discussed in Section 3.2, sustained activation of the IRE1α–TRAF2–ASK1–JNK pathway promotes tau hyperphosphorylation. Studies in a *Caenorhabditis elegans* tauopathy model further showed that XBP1s-dependent transcriptional programs alleviate tau toxicity through coordinated regulation of molecular chaperones and lipid metabolism (Waldherr et al., 2024; Waldh et al., 2019). These observations indicate that IRE1α signaling can influence tau pathology through mechanisms that are not readily explained by changes in APP processing alone.

A separate question is whether pathogenic tau itself is sufficient to activate canonical IRE1α signaling. In the rTg4510 mouse model, tau accumulation was not accompanied by activation of XBP1 splicing or other canonical unfolded protein response pathways (Pitera et al., 2019), indicating that tau pathology alone may be insufficient to engage the classical ER stress response. This finding is consistent with the predominantly cytosolic localization of tau, in contrast to proteins that accumulate within the endoplasmic reticulum and directly disrupt ER proteostasis. The available evidence therefore favors an indirect influence of IRE1α on tau pathology through downstream kinase signaling or XBP1s-dependent proteostatic programs rather than direct recognition of pathogenic tau by the canonical IRE1α activation pathway. At present, direct evidence defining these mechanisms in mammalian AD models remains limited. Resolving how IRE1α signaling intersects with tau pathology independently of amyloidogenic APP processing will be important for understanding its broader contribution to disease progression.

## 7 Conclusion

The cumulative evidence reviewed here, derived predominantly from preclinical models, supports a conceptual framework in which AD progression may be shaped not only by extracellular Aβ accumulation, but also by progressive erosion of adaptive neuronal proteostatic capacity. Within this framework, IRE1α may function as a context-dependent signaling hub whose downstream outputs are influenced by the intracellular proteostatic state of the neuron. Whether this adaptive-to-maladaptive transition occurs in the same manner in human AD remains to be established. Early activation of the IRE1α–XBP1s axis may transiently preserve protein quality control and limit amyloidogenic stress, whereas persistent proteotoxic remodeling progressively biases signaling toward chronic RIDD activity, kinase-driven APP mis-trafficking, and maladaptive stress amplification. The transition toward a sustained RIDD-dominant state may therefore represent a critical mechanistic event linking chronic ER stress to irreversible neuronal dysfunction during AD progression.

Preclinical studies in AD mouse models (Duran-Aniotz et al., 2023; Hafycz et al., 2023) suggest that restoration of adaptive XBP1s signaling may be most effective during the preclinical or early prodromal stages, when residual proteostatic buffering capacity remains biologically recoverable. Whether this stage-dependent therapeutic window extends to human AD remains to be established. Once chronic phospho-IRE1α oligomerization, calcium dyshomeostasis, mitochondrial dysfunction, and inflammatory amplification loops become self-sustaining, amyloid clearance alone may be insufficient to restore neuronal homeostasis. Within the proposed IRE1α framework, therapeutic responsiveness in AD may depend less on amyloid burden than on the extent to which adaptive IRE1α–XBP1s signaling and the associated intracellular proteostatic reserve remain intact at the time of intervention.

## Glossary

ABCA7: ATP-binding cassette transporter A7
AD: Alzheimer’s disease
ADAM10: a disintegrin and metalloproteinase 10
APOE: apolipoprotein E
APP: amyloid precursor protein
ASK1: apoptosis signal-regulating kinase 1
ATF4: activating transcription factor 4
ATF6: activating transcription factor 6
AAV: adeno-associated virus
Aβ: amyloid-beta
BACE1: beta-site APP-cleaving enzyme 1
BiP: binding immunoglobulin protein (see GRP78)
CHOP: C/EBP homologous protein
CLU: clusterin
CNS: central nervous system
CSF: cerebrospinal fluid
eIF2α: eukaryotic initiation factor 2 alpha
ER: endoplasmic reticulum
ERAD: ER-associated degradation
GRP78: glucose-regulated protein 78 (also known as BiP)
GRP94: glucose-regulated protein 94
HRD1: HMGCR degradation protein 1
HSPA5: heat shock protein family A member 5
IL-1β: interleukin-1 beta
iPSC: induced pluripotent stem cell
IRE1α: inositol-requiring enzyme 1 alpha
IXA4: IRE1/XBP1s pharmacological activator compound 4
JNK: c-Jun N-terminal kinase
KIRA6: kinase-inhibiting RNase attenuator 6
KLC: kinesin light chain
4-PBA: 4-phenylbutyric acid
MAM: mitochondria-associated ER membrane
MANF: Mesencephalic Astrocyte-Derived Neurotrophic Factor
MCI: mild cognitive impairment
NF-κB: nuclear factor kappa B
NRF1: nuclear respiratory factor 1
PDI: protein disulfide isomerase
PERK: PKR-like ER kinase
PSD-95: postsynaptic density protein 95
RIDD: regulated IRE1α-dependent decay
RQC: ribosome quality control
RyR3: ryanodine receptor 3
SEL1L: suppressor/enhancer of Lin-12-like protein 1
STF-083010: selective IRE1α RNase inhibitor
TNF-α: tumor necrosis factor alpha
TRAF2: TNF receptor-associated factor 2
TUDCA: tauroursodeoxycholic acid
UPR: unfolded protein response
UTR: untranslated region
XBP1s: spliced X-box binding protein 1
VCP: Valosin-containing protein

## References

[B1] Adams C. J. Kopp M. C. Larburu N. Nowak P. R. Ali M. M. U. (2019). Structure and molecular mechanism of ER stress signaling by the unfolded protein response signal activator IRE1. Front. Mol. Biosci. 6, 11. 10.3389/fmolb.2019.00011 30931312 PMC6423427

[B2] Ajoolabady A. Lindholm D. Ren J. Pratico D. (2022). ER stress and UPR in Alzheimer’s disease: mechanisms, pathogenesis, treatments. Cell Death Dis. 13 (8), 706. 10.1038/s41419-022-05153-5 35970828 PMC9378716

[B3] Ando K. Oishi M. Takeda S. Iijima K. Isohara T. Nairn A. C. (1999). Role of phosphorylation of Alzheimer's amyloid precursor protein during neuronal differentiation. J. Neurosci. 19 (11), 4421–4427. 10.1523/jneurosci.19-11-04421.1999 10341243 PMC6782598

[B4] Arber C. Toombs J. Lovejoy C. Ryan N. S. Paterson R. W. Willumsen N. (2020). Familial Alzheimer’s disease patient-derived neurons reveal distinct mutation-specific effects on amyloid beta. Mol. Psychiatry 25 (11), 2919–2931. 10.1038/s41380-019-0410-8 30980041 PMC7577860

[B5] Area-Gomez E. Del Carmen Lara Castillo M. Tambini M. D. Guardia-Laguarta C. de Groof A. J. Madra M. (2012). Upregulated function of mitochondria-associated ER membranes in Alzheimer disease. Embo J. 31 (21), 4106–4123. 10.1038/emboj.2012.202 22892566 PMC3492725

[B6] Belyy V. Zuazo-Gaztelu I. Alamban A. Ashkenazi A. Walter P. (2022). Endoplasmic reticulum stress activates human IRE1α through reversible assembly of inactive dimers into small oligomers. Elife 11, e74342. 10.7554/elife.74342 35730415 PMC9217129

[B7] Brase L. You S. F. D'Oliveira Albanus R. Del-Aguila J. L. Dai Y. Novotny B. C. (2023). Single-nucleus RNA-sequencing of autosomal dominant Alzheimer disease and risk variant carriers. Nat. Commun. 14 (1), 2314. 10.1038/s41467-023-37437-5 37085492 PMC10121712

[B8] Buchanan H. Mackay M. Palmer K. Tothová K. Katsur M. Platt B. (2020). Synaptic loss, ER stress and neuro-inflammation emerge late in the lateral temporal cortex and associate with progressive tau pathology in Alzheimer’s disease. Mol. Neurobiol. 57 (8), 3258–3272. 10.1007/s12035-020-01950-1 32514860 PMC7340653

[B9] Cabral-Miranda F. Tamburini G. Martinez G. Ardiles A. O. Medinas D. B. Gerakis Y. (2022). Unfolded protein response IRE1/XBP1 signaling is required for healthy Mammalian brain aging. Embo J. 41 (22), e111952. 10.15252/embj.2022111952 36314651 PMC9670206

[B10] Calvo-Rodriguez M. Hou S. S. Snyder A. C. Kharitonova E. K. Russ A. N. Das S. (2020). Increased mitochondrial calcium levels associated with neuronal death in a mouse model of Alzheimer’s disease. Nat. Commun. 11 (1), 2146. 10.1038/s41467-020-16074-2 32358564 PMC7195480

[B11] Casas-Tinto S. Zhang Y. Sanchez-Garcia J. Gomez-Velazquez M. Rincon-Limas D. E. Fernandez-Funez P. (2011). The ER stress factor XBP1s prevents amyloid-beta neurotoxicity. Hum. Mol. Genet. 20 (11), 2144–2160. 10.1093/hmg/ddr100 21389082 PMC3090193

[B12] Chu B. Li M. Cao X. Li R. Jin S. Yang H. (2021). IRE1α-XBP1 affects the mitochondrial function of Aβ25-35-Treated SH-SY5Y cells by regulating mitochondria-associated endoplasmic reticulum membranes. Front. Cell Neurosci. 15, 614556. 10.3389/fncel.2021.614556 33841100 PMC8027129

[B13] Ciechanover A. Kwon Y. T. (2017). Protein quality control by molecular chaperones in neurodegeneration. Front. Neurosci. 11, 185. 10.3389/fnins.2017.00185 28428740 PMC5382173

[B14] Crawford K. Leonenko G. Baker E. Grozeva D. Lan-Leung B. Holmans P. (2023). Golgi apparatus, endoplasmic reticulum and mitochondrial function implicated in Alzheimer’s disease through polygenic risk and RNA sequencing. Mol. Psychiatry 28 (3), 1327–1336. 10.1038/s41380-022-01926-8 36577842 PMC10005937

[B15] Duran-Aniotz C. Cornejo V. H. Espinoza S. Ardiles Á. O. Medinas D. B. Salazar C. (2017). IRE1 signaling exacerbates Alzheimer’s disease pathogenesis. Acta Neuropathol. 134 (3), 489–506. 10.1007/s00401-017-1694-x 28341998

[B16] Duran-Aniotz C. Poblete N. Rivera-Krstulovic C. Ardiles Á. O. Díaz-Hung M. L. Tamburini G. (2023). The unfolded protein response transcription factor XBP1s ameliorates Alzheimer’s disease by improving synaptic function and proteostasis. Mol. Ther. 31 (7), 2240–2256. 10.1016/j.ymthe.2023.03.028 37016577 PMC10362463

[B17] Gerakis Y. Hetz C. (2018). A decay of the adaptive capacity of the unfolded protein response exacerbates Alzheimer’s disease. Neurobiol. Aging 63, 162–164. 10.1016/j.neurobiolaging.2017.09.012 29042130

[B18] Gerakis Y. Dunys J. Bauer C. Checler F. (2016). Aβ42 oligomers modulate β-secretase through an XBP-1s-dependent pathway involving HRD1. Sci. Rep. 6, 37436. 10.1038/srep37436 27853315 PMC5112606

[B19] Ghosh R. Wang L. Wang E. S. Perera B. G. Igbaria A. Morita S. (2014). Allosteric inhibition of the IRE1α RNase preserves cell viability and function during endoplasmic reticulum stress. Cell 158 (3), 534–548. 10.1016/j.cell.2014.07.002 25018104 PMC4244221

[B20] Goetzl E. J. Boxer A. Schwartz J. B. Abner E. L. Petersen R. C. Miller B. L. (2015). Altered lysosomal proteins in neural-derived plasma exosomes in preclinical Alzheimer disease. Neurology 85 (1), 40–47. 10.1212/WNL.0000000000001702 26062630 PMC4501943

[B21] Gomez J. A. Rutkowski D. T. (2016). Experimental reconstitution of chronic ER stress in the liver reveals feedback suppression of BiP mRNA expression. Elife 5. 10.7554/eLife.20390 27938665 PMC5179193

[B22] Gómora-García J. C. Gerónimo-Olvera C. Pérez-Martínez X. Massieu L. (2021). IRE1α RIDD activity induced under ER stress drives neuronal death by the degradation of 14-3-3 θ mRNA in cortical neurons during glucose deprivation. Cell Death Discov. 7 (1), 131. 10.1038/s41420-021-00518-9 34083523 PMC8175356

[B23] Grandjean J. M. D. Madhavan A. Cech L. Seguinot B. O. Paxman R. J. Smith E. (2020). Pharmacologic IRE1/XBP1s activation confers targeted ER proteostasis reprogramming. Nat. Chem. Biol. 16 (10), 1052–1061. 10.1038/s41589-020-0584-z 32690944 PMC7502540

[B24] Hafycz J. M. Strus E. Sengupta K. Naidoo N. N. (2023). Early and late chaperone intervention therapy boosts XBP1s and ADAM10, restores proteostasis, and rescues learning in Alzheimer's disease mice. Aging Biol. 1, 20230017. 10.59368/agingbio.20230017 41788816 PMC12959931

[B25] Hampel H. Vassar R. De Strooper B. Hardy J. Willem M. Singh N. (2021). The β-Secretase BACE1 in Alzheimer's disease. Biol. Psychiatry 89 (8), 745–756. 10.1016/j.biopsych.2020.02.001 32223911 PMC7533042

[B26] Hashimoto S. Saido T. C. (2018). Critical review: involvement of endoplasmic reticulum stress in the aetiology of Alzheimer's disease. Open Biol. 8 (4). 10.1098/rsob.180024 29695619 PMC5936719

[B27] Hébert S. S. Horré K. Nicolaï L. Papadopoulou A. S. Mandemakers W. Silahtaroglu A. N. (2008). Loss of microRNA cluster miR-29a/b-1 in sporadic Alzheimer’s disease correlates with increased BACE1/beta-secretase expression. Proc. Natl. Acad. Sci. U. S. A. 105 (17), 6415–6420. 10.1073/pnas.0710263105 18434550 PMC2359789

[B28] Hetz C. Papa F. R. (2018). The unfolded protein response and cell fate control. Mol. Cell 69 (2), 169–181. 10.1016/j.molcel.2017.06.017 29107536

[B29] Hetz C. Thielen P. Matus S. Nassif M. Court F. Kiffin R. (2009). XBP-1 deficiency in the nervous system protects against amyotrophic lateral sclerosis by increasing autophagy. Genes Dev. 23 (19), 2294–2306. 10.1101/gad.1830709 19762508 PMC2758741

[B30] Hetz C. Zhang K. Kaufman R. J. (2020). Mechanisms, regulation and functions of the unfolded protein response. Nat. Rev. Mol. Cell Biol. 21 (8), 421–438. 10.1038/s41580-020-0250-z 32457508 PMC8867924

[B31] Hollien J. Lin J. H. Li H. Stevens N. Walter P. Weissman J. S. (2009). Regulated Ire1-dependent decay of messenger RNAs in Mammalian cells. J. Cell Biol. 186 (3), 323–331. 10.1083/jcb.200903014 19651891 PMC2728407

[B32] Hoozemans J. J. van Haastert E. S. Nijholt D. A. Rozemuller A. J. Eikelenboom P. Scheper W. (2009). The unfolded protein response is activated in pretangle neurons in Alzheimer’s disease hippocampus. Am. J. Pathol. 174 (4), 1241–1251. 10.2353/ajpath.2009.080814 19264902 PMC2671357

[B33] Jiang S. Srikanth M. Serpe R. Yavari S. Gaur P. Collins G. A. (2025). Early proteasome downregulation and dysfunction drive proteostasis failure in Alzheimer’s disease. Brain 148 (12), 4372–4388. 10.1093/brain/awaf222 40488453 PMC13370123

[B34] Korovesis D. Rufo N. Derua R. Agostinis P. Verhelst S. H. L. (2020). Kinase photoaffinity labeling reveals low selectivity profile of the IRE1 targeting Imidazopyrazine-Based KIRA6 inhibitor. ACS Chem. Biol. 15 (12), 3106–3111. 10.1021/acschembio.0c00802 33290055

[B35] Le Thomas A. Ferri E. Marsters S. Harnoss J. M. Lawrence D. A. Zuazo-Gaztelu I. (2021). Decoding non-canonical mRNA decay by the endoplasmic-reticulum stress sensor IRE1α. Nat. Commun. 12 (1), 7310. 10.1038/s41467-021-27597-7 34911951 PMC8674358

[B36] Li Q. Liu T. Yang S. Zhang Z. (2019). Upregulation of miR-34a by inhibition of IRE1α has protective effect against Aβ-Induced injury in SH-SY5Y cells by targeting Caspase-2. Oxid. Med. Cell Longev. 2019, 2140427. 10.1155/2019/2140427 31281568 PMC6589233

[B37] Li X. Sun S. Appathurai S. Sundaram A. Plumb R. Mariappan M. (2020). A molecular mechanism for turning off IRE1α signaling during endoplasmic reticulum stress. Cell Rep. 33 (13), 108563. 10.1016/j.celrep.2020.108563 33378667 PMC7809255

[B38] Li X. Feng X. Sun X. Hou N. Han F. Liu Y. (2022). Global, regional, and national burden of Alzheimer’s disease and other dementias, 1990-2019. Front. Aging Neurosci. 14, 937486. 10.3389/fnagi.2022.937486 36299608 PMC9588915

[B39] Li Y. Liu D. Zhang X. Rimal S. Lu B. Li S. (2024). RACK1 and IRE1 participate in the translational quality control of amyloid precursor protein in Drosophila models of Alzheimer’s disease. J. Biol. Chem. 300 (3), 105719. 10.1016/j.jbc.2024.105719 38311171 PMC10907166

[B40] Liang Y. Ye C. Chen Y. Chen Y. Diao S. Huang M. (2021). Berberine improves behavioral and cognitive deficits in a mouse model of Alzheimer's disease via regulation of β-Amyloid production and endoplasmic reticulum stress. ACS Chem. Neurosci. 12 (11), 1894–1904. 10.1021/acschemneuro.0c00808 33983710

[B41] Mahameed M. Wilhelm T. Darawshi O. Obiedat A. Tommy W. S. Chintha C. (2019). The unfolded protein response modulators GSK2606414 and KIRA6 are potent KIT inhibitors. Cell Death Dis. 10 (4), 300. 10.1038/s41419-019-1523-3 30931942 PMC6443726

[B42] Marcora M. S. Belfiori-Carrasco L. F. Bocai N. I. Morelli L. Castaño E. M. (2017). Amyloid-β42 clearance and neuroprotection mediated by X-box binding protein 1 signaling decline with aging in the Drosophila brain. Neurobiol. Aging 60, 57–70. 10.1016/j.neurobiolaging.2017.08.012 28917667

[B43] Maurel M. Chevet E. Tavernier J. Gerlo S. (2014). Getting RIDD of RNA: IRE1 in cell fate regulation. Trends Biochem. Sci. 39 (5), 245–254. 10.1016/j.tibs.2014.02.008 24657016

[B44] Mórotz G. M. Glennon E. B. Greig J. Lau D. H. W. Bhembre N. Mattedi F. (2019). Kinesin light chain-1 serine-460 phosphorylation is altered in Alzheimer’s disease and regulates axonal transport and processing of the amyloid precursor protein. Acta Neuropathol. Commun. 7 (1), 200. 10.1186/s40478-019-0857-5 31806024 PMC6896704

[B45] Naidoo N. (2009). ER and aging-protein folding and the ER stress response. Ageing Res. Rev. 8 (3), 150–159. 10.1016/j.arr.2009.03.001 19491040

[B46] Nunes A. F. Amaral J. D. Lo A. C. Fonseca M. B. Viana R. J. Callaerts-Vegh Z. (2012). TUDCA, a bile acid, attenuates amyloid precursor protein processing and amyloid-β deposition in APP/PS1 mice. Mol. Neurobiol. 45 (3), 440–454. 10.1007/s12035-012-8256-y 22438081

[B47] Pitera A. P. Asuni A. A. O'Connor V. Deinhardt K. (2019). Pathogenic tau does not drive activation of the unfolded protein response. J. Biol. Chem. 294 (25), 9679–9688. 10.1074/jbc.RA119.008263 31053641 PMC6597832

[B48] Poe C. Youngblood C. Hodge K. Kemp K. (2019). Treatment of established TH2 cells with 4μ8c, an inhibitor of IRE1α, blocks IL-5 but not IL-4 secretion. BMC Immunol. 20 (1), 3. 10.1186/s12865-018-0283-7 30630412 PMC6327572

[B49] Reinhardt S. Schuck F. Grösgen S. Riemenschneider M. Hartmann T. Postina R. (2014). Unfolded protein response signaling by transcription factor XBP-1 regulates ADAM10 and is affected in Alzheimer’s disease. Faseb J. 28 (2), 978–997. 10.1096/fj.13-234864 24165480

[B50] Rufo N. Korovesis D. Van Eygen S. Derua R. Garg A. D. Finotello F. (2022). Stress-induced inflammation evoked by immunogenic cell death is blunted by the IRE1α kinase inhibitor KIRA6 through HSP60 targeting. Cell Death Differ. 29 (1), 230–245. 10.1038/s41418-021-00853-5 34453119 PMC8738768

[B51] Salminen A. Kauppinen A. Suuronen T. Kaarniranta K. Ojala J. (2009). ER stress in Alzheimer’s disease: a novel neuronal trigger for inflammation and Alzheimer’s pathology. J. Neuroinflammation 6, 41. 10.1186/1742-2094-6-41 20035627 PMC2806266

[B52] Scheper W. Hoozemans J. J. (2015). The unfolded protein response in neurodegenerative diseases: a neuropathological perspective. Acta Neuropathol. 130 (3), 315–331. 10.1007/s00401-015-1462-8 26210990 PMC4541706

[B53] Sims J. R. Zimmer J. A. Evans C. D. Lu M. Ardayfio P. Sparks J. (2023). Donanemab in early symptomatic Alzheimer disease: the TRAILBLAZER-ALZ 2 randomized clinical trial. Jama 330 (6), 512–527. 10.1001/jama.2023.13239 37459141 PMC10352931

[B54] van Dyck C. H. Swanson C. J. Aisen P. Bateman R. J. Chen C. Gee M. (2023). Lecanemab in early Alzheimer’s disease. N. Engl. J. Med. 388 (1), 9–21. 10.1056/nejmoa2212948 36449413

[B55] Vidal R. L. Figueroa A. Court F. A. Thielen P. Molina C. Wirth C. (2012). Targeting the UPR transcription factor XBP1 protects against Huntington’s disease through the regulation of FoxO1 and autophagy. Hum. Mol. Genet. 21 (10), 2245–2262. 10.1093/hmg/dds040 22337954 PMC3335312

[B56] Volmer R. van der Ploeg K. Ron D. (2013). Membrane lipid saturation activates endoplasmic reticulum unfolded protein response transducers through their transmembrane domains. Proc. Natl. Acad. Sci. U. S. A. 110 (12), 4628–4633. 10.1073/pnas.1217611110 23487760 PMC3606975

[B57] Waldherr S. M. Strovas T. J. Vadset T. A. Liachko N. F. Kraemer B. C. (2019). Constitutive XBP-1s-mediated activation of the endoplasmic reticulum unfolded protein response protects against pathological tau. Nat. Commun. 10 (1), 4443. 10.1038/s41467-019-12070-3 31570707 PMC6768869

[B58] Waldherr S. M. Han M. Saxton A. D. Vadset T. A. McMillan P. J. Wheeler J. M. (2024). Endoplasmic reticulum unfolded protein response transcriptional targets of XBP-1s mediate rescue from tauopathy. Commun. Biol. 7 (1), 903. 10.1038/s42003-024-06570-2 39060347 PMC11282107

[B59] Wang W. X. Rajeev B. W. Stromberg A. J. Ren N. Tang G. Huang Q. (2008). The expression of microRNA miR-107 decreases early in Alzheimer’s disease and may accelerate disease progression through regulation of beta-site amyloid precursor protein-cleaving enzyme 1. J. Neurosci. 28 (5), 1213–1223. 10.1523/JNEUROSCI.5065-07.2008 18234899 PMC2837363

[B60] Zhang Y. Chen X. Chen L. Shao M. Zhu W. Xing T. (2024). Increased expression of mesencephalic astrocyte-derived neurotrophic factor (MANF) contributes to synapse loss in Alzheimer's disease. Mol. Neurodegener. 19 (1), 75. 10.1186/s13024-024-00771-3 39425207 PMC11490049

